# Free radical-mediated acetaldehyde formation by model reactions of dietary components: effects of meat, wine, cooking oil and coffee

**DOI:** 10.1186/s41021-021-00201-6

**Published:** 2021-07-09

**Authors:** Hiroshi Kasai, Kazuaki Kawai

**Affiliations:** 1 Department of Environmental Oncology, Institute of Industrial Ecological Sciences, University of Occupational and Environmental Health, 1-1 Iseigaoka, Yahatanishi-ku, Fukuoka 807-8555 Kitakyushu, Japan; 2 Center for Stress-related Disease Control and Prevention, University of Occupational and Environmental Health, 1-1 Iseigaoka, Yahatanishi-ku, Fukuoka 807-8555 Kitakyushu, Japan

**Keywords:** Acetaldehyde, Gastric cancer, Colorectal cancer, Heme, Myoglobin, Linoleate, Polyphenol, Meat, Wine, Coffee

## Abstract

**Background:**

Alcohol consumption and the ingestion of red meat and oxidized cooking oil are risk factors of gastric and colorectal cancers. We reported that acetaldehyde (AcAld) is generated from Heme/Mb/Meat-Linoleate-EtOH model reaction mixtures, and thus could be a new plausible mechanism for the carcinogenesis (Kasai and Kawai, ACS Omega, 2021).

**Results:**

In this study, we investigated the effects of wine and coffee, in addition to meat components, on this reaction. Depending on the conditions, such as pH, reaction time and choice of free hemin, myoglobin (Mb), as well as meat extracts (raw meat, baked meat, salami), wine and coffee enhanced AcAld formation. Polyphenols in red wine and coffee may stimulate AcAld formation by acting as pro-oxidants in the presence of Heme/Mb/Meat. In a model reaction of Mb + EtOH + H_2_O_2_, we observed time-dependent AcAld formation. In support of these *in vitro* data, after the consumption of a red meat-rich diet with red wine, the fecal AcAld level significantly increased as compared to the levels associated with a diet of fish + wine, or red meat without alcohol.

**Conclusions:**

These results suggested that AcAld generation from dietary components may be an important mechanism of gastrointestinal tract carcinogenesis.

## Introduction

Epidemiological studies are a powerful method to identify the causes of cancer. Alcohol drinking and the consumption of red meat, especially processed meat, are risk factors of gastric and colorectal cancers, as revealed by epidemiological studies [1–4]. Fried foods and reuse of cooking oil also increase the incidence of these cancers [5–7]. The most frequent mutation in the p53 tumor suppressor gene observed in these cancers is GC→AT in specific positions with GpC sequences, which are often methylated [8], and the characteristics of the mutation spectrum and the hot spots are similar to those found in the p53 gene, induced by AcAld in a yeast functional assay system [9, 10]. Based on this information from epidemiological and genomic studies, it is also important to clarify the carcinogenesis mechanisms by a chemical approach [11]. After extensive studies focusing on AcAld, we found its efficient formation in model reactions containing three components of these risk factors, Heme/Mb/Meat, linoleate and alcohol, but not by the combinations of any two [12]. As a mechanism, linoleate hydroperoxide formation and decomposition in the presence of myoglobin (or heme) to generate an OH radical seem to be involved in the ethanol to acetaldehyde conversion (Scheme). This may represent a new plausible mechanism of gastric and colorectal carcinogenesis.

**Scheme 1:**
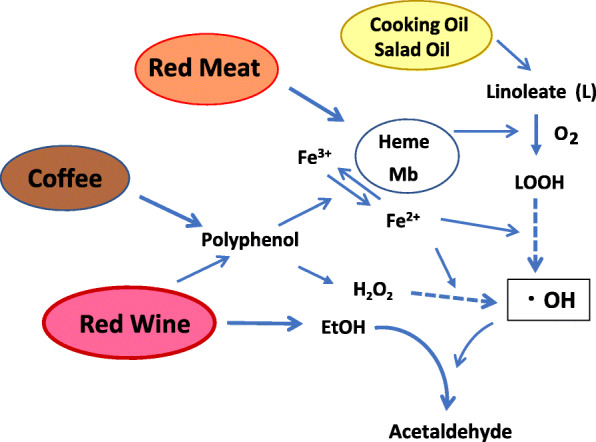
Free radical-mediated AcAld formation from dietary components

AcAld is an environmental mutagen and considerable amounts are present in ordinary foods and beverages [13]. It is a human carcinogen (IARC, group 1), especially in relation to alcohol consumption [14]. Alcohol is metabolized to AcAld by alcohol dehydrogenase (ADH) enzymes, and then to acetic acid by aldehyde dehydrogenase (ALDH). High incidences of oral, esophageal, stomach and colon cancers have been observed in ALDH-deficient subjects [1]. In relation to red meat intake, N-nitroso compounds, such as N-nitroso-glucosyl-glycine, and aldehydic compounds, such as 4-hydroxy-2-nonenal and malonaldehyde, generated by heme-induced lipid peroxidation, have been suggested as possible carcinogenesis mechanisms [15], but the role of AcAld has not been described so far. In this study, we investigated the effects of red wine and coffee on the formation of AcAld in the model reactions, because meat, wine and coffee are often consumed together in ordinary daily dietary habits. Various model reactions were examined at pH 4.5 and pH 7.4, as representative pH values of the gastric juices of normal and high-risk groups, respectively (further details in the Discussion). The experiments at pH 7.4 also correspond to the pH of the colonic contents of colon cancer high-risk groups. As an initial condition of digestion in the healthy stomach, the reaction of meat + wine + coffee was examined at pH 3.5. We also investigated whether various diet combinations affect the fecal AcAld levels, in relation to colorectal carcinogenesis.

## Experimental section

### Materials

Hemin was purchased from Sigma-Aldrich Chemical Co., USA. Tween 20 was obtained from ICN Biochemicals, Inc., USA. Horse myoglobin (Mb) was procured from SERVA Electrophoresis GmbH, Germany. Methyl linoleate (MLA) was purchased from Tokyo Chemical Industry Co., Ltd. Japan. 2,4-Dinitrophenylhydrazine (DNPH), ethanol (99.5 %), and hydrogen peroxide (30 %) were obtained from Wako Pure Chemical Industries, Ltd., Japan. Beef, salami, red wine (13.5 % alcohol), and instant coffee were purchased in a grocery store.

### Preparation of meat homogenates

Meat was baked in a pan without cooking oil until it was browned, as in the usual preparation. Portions (0.7 g) of raw and baked meat, or salami were cut into small pieces and homogenized in water (5 ml) containing Tween 20 (10 µl, 0.2 %), with a Polytron PT10-35 (Kinematica, Switzerland) for 30 s at room temperature. The homogenates were divided into 500 µl aliquots in Eppendorf tubes (2 ml) and kept in a freezer at -20 °C until use.

### Reaction of hemin-MLA-EtOH/Wine

Hemin was dissolved in 20 mM NaOH (2.17 mg/ml). For the ethanol reaction mixture, the hemin solution (23 µl; final concentration, 160 µM), ethyl acetate (50 µl), MLA (20 µl), ethanol (50 µl), and 2 M sodium acetate buffer (40 µl, pH 4.5), and water (360 µL) were mixed in an Eppendorf tube (2 ml). The tube was capped and vigorously shaken to produce a homogeneous emulsion at 37 °C. The reaction was continued for 4 h. For the reaction at pH 7.4, 2 M phosphate buffer (pH 7.4) was used instead of the acetate buffer. For the wine reaction mixture, the hemin solution (23 µl; final concentration, 160 µM), ethyl acetate (50 µl), MLA (20 µl), wine (369 µl), 2 M buffer (40 µl), and water (41 µL) were mixed.

### Reaction of meat-MLA-EtOH/Wine

MLA (20 µl), 2 M phosphate buffer (pH 7.4) (55 µl), and ethanol (23 µl, plus 147 µL water) or wine (170 µL) were added to the meat homogenates (500 µl) in an Eppendorf tube (2 ml). The tube was capped and vigorously shaken to produce a homogeneous emulsion at 37 °C. In these reactions, the approximate heme concentration was 50 µM, based on the assumption that meats contain 1 % Mb.

### Reaction of baked meat-wine-coffee

Coffee was prepared by dissolving 2 g of instant coffee powder in 140 mL of hot water. Baked meat homogenate (300 µL, 50 µM as heme), red wine (150 µL) and coffee (150 µL) were mixed with 1 M NaH_2_PO_4_ (300 µL), and the final pH was adjusted to 3.5 by adding 2 N HCl. The tube was capped and vigorously shaken to produce a homogeneous emulsion at 37 °C. For the reaction of two components (baked meat, wine), water (150 µL) was added instead of coffee. For the reaction without meat and coffee, 450 µL of water was added instead.

### Reaction of Mb-EtOH-H_2_O_2_

A 500 µl aliquot of the Mb solution (1.3 mg/ml water plus 2 µl Tween 20) (final concentration, 60 µM) was mixed with ethanol (50 µl), H_2_O_2_ (2.7 µL, final concentration 40 mM), and 2 M phosphate buffer (pH 7.4) (55 µl) in an Eppendorf tube (2 ml). The tube was capped and incubated at 37 °C.

### Analysis of AcAld in the reaction mixture

AcAld was analyzed by the previously described method [12]. Briefly, after centrifugation of the emulsion reaction mixture, 10 µl of the supernatant (or water for blank) was mixed with 100 µl of 0.2 M sodium acetate (pH 4.5) and 100 µl of 2,4-dinitrophenylhydrazine solution in acetonitrile (1.25 mg/ml), and reacted for 3 min at room temperature (23 °C). After centrifugation, a 50 µl portion of the supernatant was immediately injected into an HPLC column (CAPCELL PAK C18 MG II, 3 μm, 4.6 mm x 150 mm, Shiseido Fine Chemicals, Japan) connected with a photodiode array UV detector (Hewlett-Packard 1100 HPLC Detection System). The following linear gradient of acetonitrile concentration in 10 mM ammonium formate was used: 0–15 min, 50–100 %; 15–20 min, 100 %. The elution speed was 0.8 ml/min. The blank value was subtracted from each analysis value of the reaction mixture. The AcAld concentration was determined based on the calibration curve.

### LC/MS

The AcAld-DNPH from feces was identified by HPLC coupled to a hybrid quadrupole-Orbitrap mass spectrometer (Q Exactive Focus, Thermo Fisher Scientific, Waltham, MA) with negative-ion ESI-MS. The sample separation was achieved on an Acclaim 120 C18 column (2.1 mm x 50 mm, 3 μm, Thermo Fisher Scientific, Waltham, MA) with a flow rate of 0.3 ml/min and a column temperature of 30 °C. Mobile phase A was 10 mM ammonium formate and mobile phase B was acetonitrile. The percentage of solvent B changed as follows: 0–2 min, 40 %; 2–10 min, 40–90 % (linear gradient). The injection volumes for the measurements were 5 µl. The AcAld-DNPH (C_8_H_8_N_4_O_4_) was identified using the extracted ion chromatogram of *m*/*z* 223.04728 [M-H]^−^.

### Analysis of AcAld in feces

A subject (male, age 73 at the time of sample collection, Asian flusher with low ALDH, body weight 51 kg) ingested three different diets: (1) meat plus wine, (2) fish plus wine, and (3) meat plus non-alcohol beer, for three consecutive days. In diet-1, the total amounts of meat (salami, sausage, etc., at breakfast, and red beef at lunch and dinner) and red wine were 200–250 g and 120–150 mL per day, respectively. Red wine was consumed during dinner. During the meat challenge and other diet periods, moderate amounts of bread, rice, yoghurt, vegetables, fruits, tea and coffee, etc., were ingested as usual. In diet-2, meat was replaced by white fish or chicken tenders, and the alcohol (red wine) consumption was the same as in diet-1. Diet-3 was the same as diet-1, except that the wine was replaced by non-alcoholic beer. On the second, third, and fourth days from the start of each diet, fecal samples were collected from 3 to 4 different positions in the morning feces, and immediately frozen and kept at -20 °C. For the AcAld analysis, an 80 µL portion of the feces was dispersed within a cold mixture of DNPH solution (800 µL) and acetate buffer (800 µL) to make emulsions during 3 min of vigorous mixing and occasional crushing of feces by toothpicks. The reaction mixture was kept at 5 °C for 20 min and then centrifuged, and a 50 µL portion of the supernatant was injected into the HPLC apparatus. Conditions for HPLC were the same as above. The study was approved by the University of Occupational and Environmental Health Ethics Committee (R3-005). Written informed consent was obtained from the subject.

## Results

### Effect of red wine on AcAld formation in model reactions

**Fig. 1:**
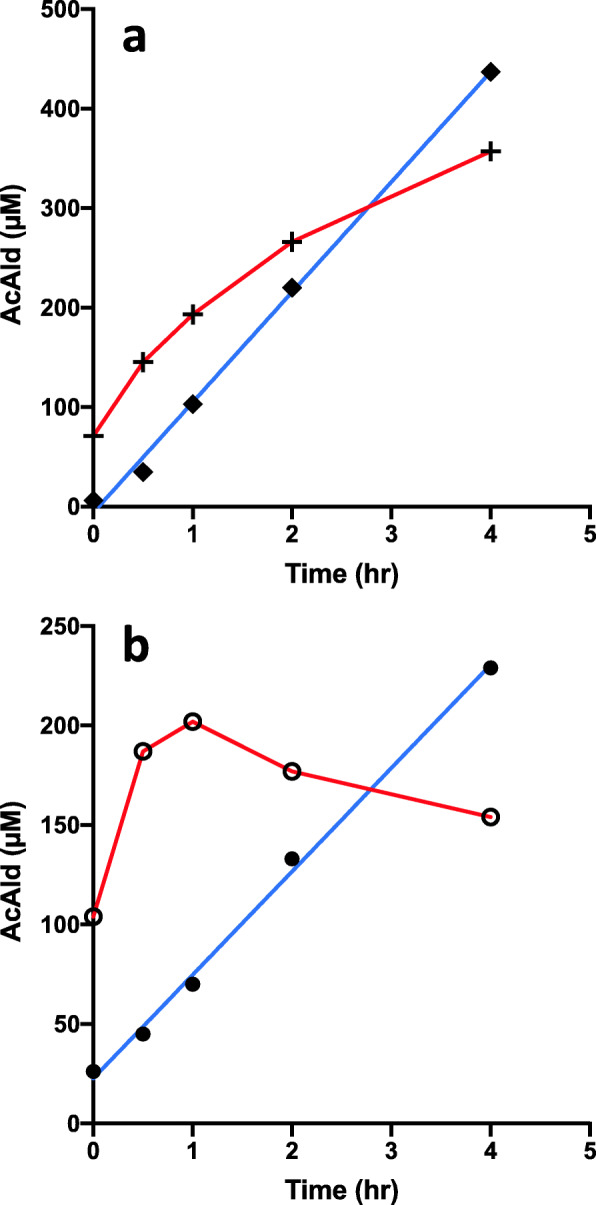
Formation of AcAld in Hemin-MLA-ethanol/wine mixtures. **a** (◆**–––**◆) Hemin + MLA + EtOH, pH 4.5; (**+–––+**) Hemin + MLA + Wine, pH 4.5. **b** (**●–––●**) Hemin + MLA + EtOH, pH 7.4; (**○–––○**) Hemin + MLA + Wine, pH 7.4

Model systems of dietary components, containing meat/Mb/hemin, MLA, and EtOH/wine, were reacted in an emulsion at pH 7.4 or 4.5. When the Hemin-MLA-EtOH- and Hemin-MLA-Wine-reactions in emulsion were compared at pH 7.4, higher AcAld generation was observed in the mixture with wine during the initial 2 h as compared to those with ethanol (Fig. 1b). At 4 h, slight inhibition was observed. The stimulation of AcAld generation by wine was not observed at pH 4.5 (Fig. 1a). At time zero, considerable amounts of AcAld (70–100 µM) were detected, because wine itself contains AcAld.

**Fig. 2:**
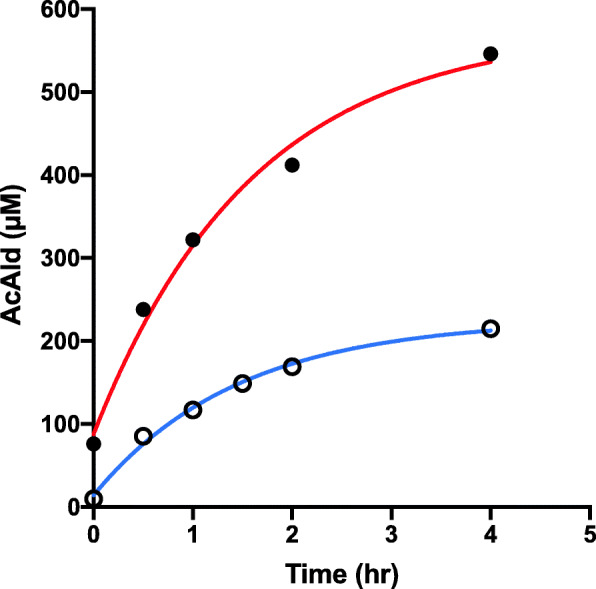
Formation of AcAld in Salami-MLA-ethanol/wine mixtures. (**●–––●**) Salami + MLA + Wine, pH 7.4; (**○–––○**) Salami + MLA + EtOH, pH 7.4

**Fig. 3:**
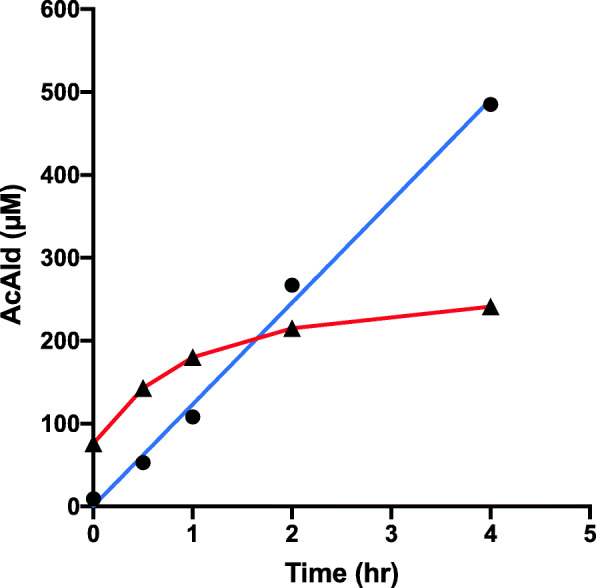
Formation of AcAld in Raw meat-MLA-ethanol/wine mixtures. (**●–––●**) Raw meat + MLA + EtOH, pH 7.4; (**▲–––▲**) Raw Meat + MLA + Wine, pH 7.4

**Fig. 4:**
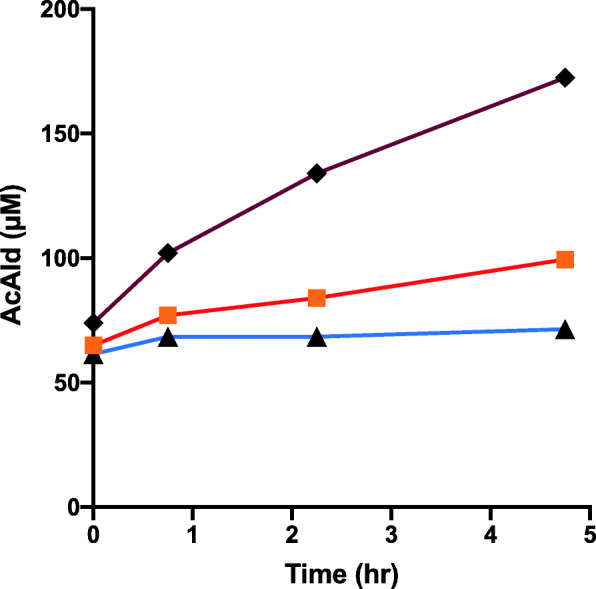
Formation of AcAld in Baked meat-Wine-Coffee mixtures. (◆**–––**◆) Baked meat + Wine + Coffee, pH 3.5; (**■––––■**) Baked meat + Wine, pH 3.5; (**▲–––▲**) Wine, pH 3.5

**Fig. 5:**
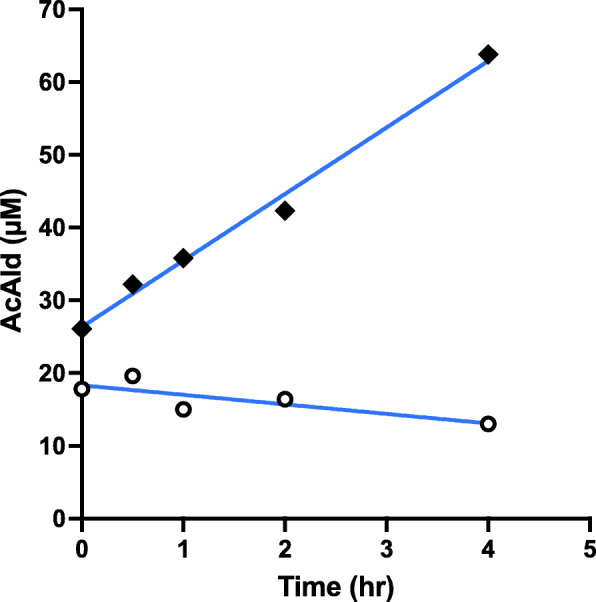
Formation of AcAld in Mb + EtOH + H_2_O_2_ mixtures (pH 7.4). (◆**–––**◆) With H_2_O_2_; (**○–––○**) without H_2_O_2_ (control)

In the salami-containing reaction, AcAld formation was strongly stimulated by wine as compared to EtOH at pH 7.4 (Fig. 2). When the reaction with raw meat was examined, wine inhibited AcAld formation, especially in the latter period (Fig. 3). In the reaction of baked meat with wine and coffee, AcAld formation was stimulated at the stomach condition of pH 3.5 (Fig. 4), without MLA. In a model reaction of Mb + EtOH + H_2_O_2_ (pH 7.4), time-dependent AcAld formation was observed (Fig. 5). In this reaction, background levels of AcAld were detected at time 0 and in the control experiments, mainly due to the presence of trace amounts of AcAld in the ethanol reagent.

### Increase of fecal AcAld after red meat plus wine diet

**Fig. 6:**
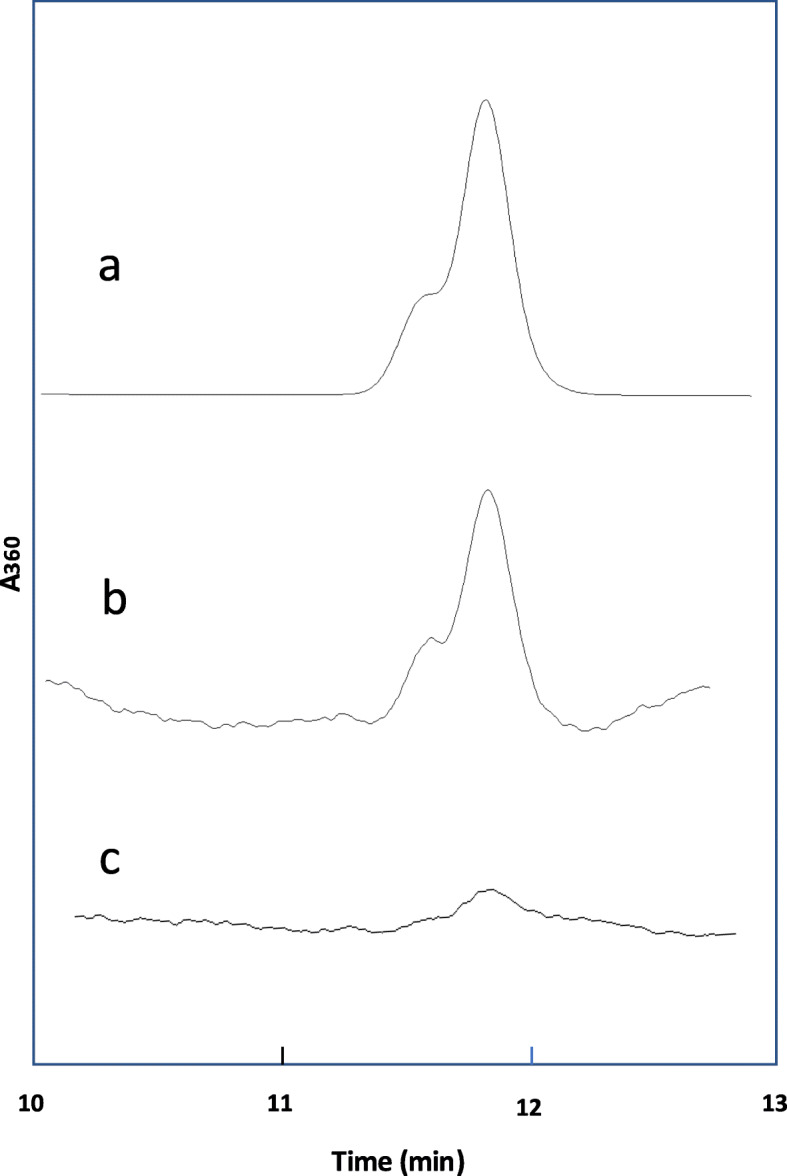
HPLC analysis of AcAld in feces derivatized with 2,4-dinitrophenyl hydrazine (DNPH). **a** Standard, **b** feces reaction mixture, **c** blank

**Fig. 7:**
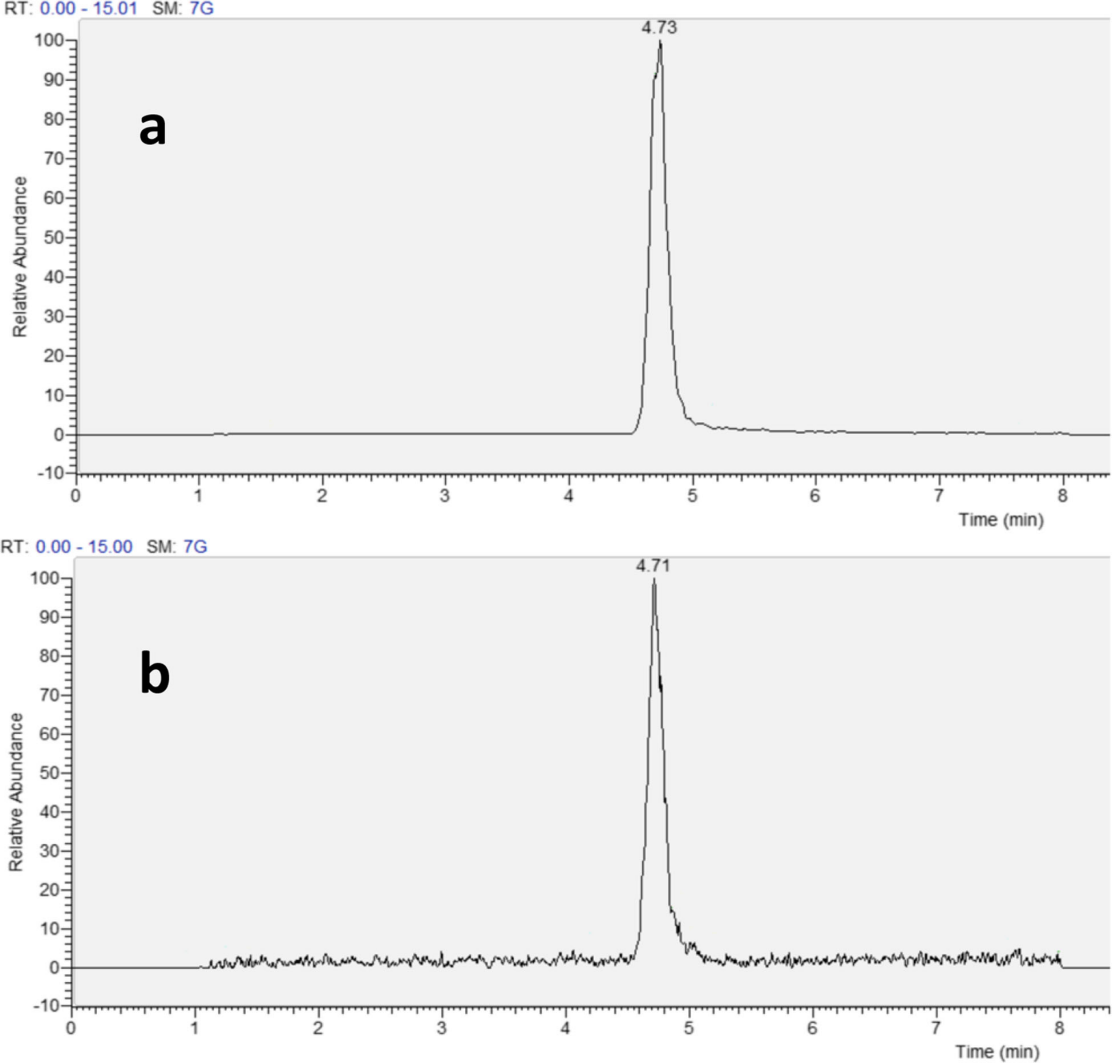
Confirmation of AcAld-DNPH peak by LC/MS (extracted ion chromatogram of *m*/*z* 223.04728 [M-H]^−^). **a** Standard, **b** AcAld-DNPH peak fraction obtained by HPLC

The chromatograms of the analysis of AcAld in feces are shown in Fig. 6. The hydrazone products produced by the reaction of AcAld with DNPH were eluted as a mixture of E- and Z-stereoisomers, as detected by the UV absorbance at 360 nm, and quantified against the standard. The retention time and UV spectrum of the product in HPLC were identical to those of the standard AcAld treated with DNPH. The identity of the AcAld-DNPH derivative was further confirmed by LC-MS (Fig. 7).

**Fig. 8:**
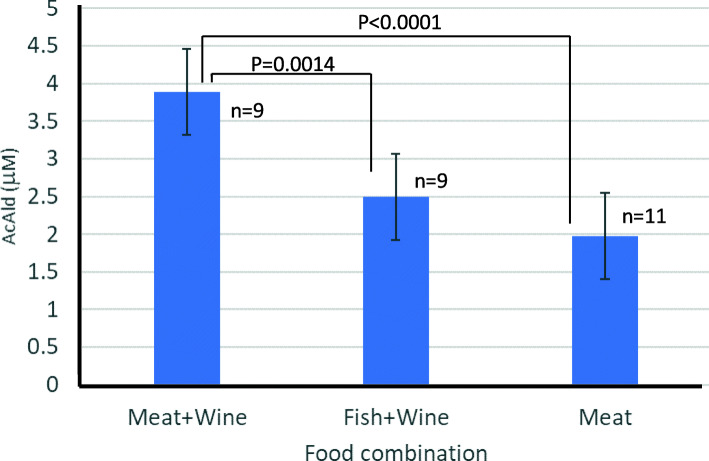
Fecal AcAld levels (mean ± SD) after different dietary combinations

After the consumption of the red meat-rich diet with wine, the fecal AcAld level significantly increased, as compared to the diets of fish + wine, or red meat without alcohol (Fig. 8). Although the AcAld level did not reach the mutagenic concentration, the results suggest that the AcAld enhancing effect observed in the model reaction could occur *in vivo* by the combination of red meat and wine.

## Discussion

Many papers have reported that wine has cancer-preventive effects, due to the high concentration of polyphenols [16]. However, heavy drinkers of wine have high incidences of gastric cancer, as revealed by epidemiological studies in France, Portugal and Paraguay [17–19]. Prospective studies in large populations demonstrated that persons who preferred wine were more likely to develop colon cancer [20, 21]. Processed meats such as sausage, ham and salami are suspected to be higher risk factors for both gastric and colon cancers than red meat [2–4]. Other epidemiological studies suggested that the co-consumption of alcohol and red meat synergistically increases the colon cancer risk [22, 23]. The effect of coffee consumption on cancer risk was also extensively studied; however, the results were controversial [24]. Coffee is a risk factor for gastric cancer [25] and colorectal cancer [26]. Because wine, meat, coffee, etc. are often consumed together in western dietary habits, it may be difficult to distinguish the effect of each individual factor by epidemiological methods [27], or to assess their combined effects on cancer induction. Our results support the hypothesis that wine and coffee enhance the gastric and colorectal carcinogenesis promoted by red meat.

**Fig. 9:**
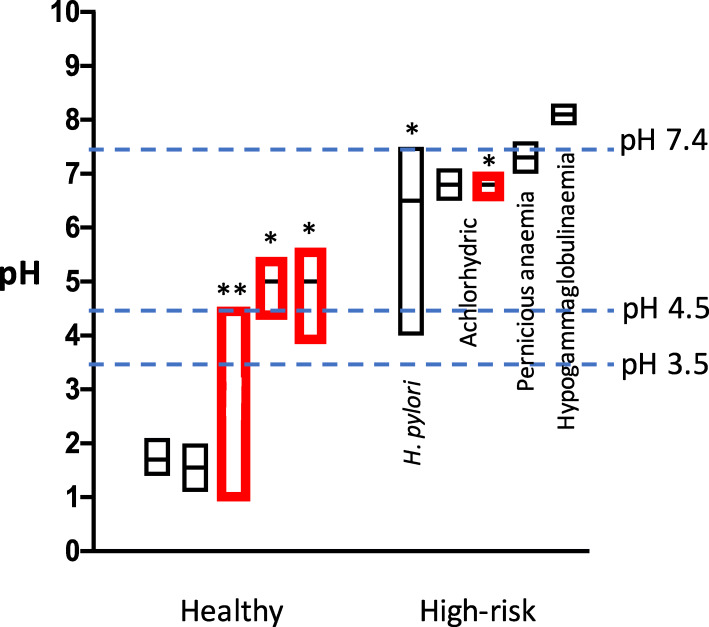
Comparison of pH ranges of gastric juice between healthy and high-risk groups. Black boxes show fasted pH, and red boxes show pH after meal. Mean ± SD are shown. *Middle bar represents median value. **Only pH range is shown

**Fig. 10:**
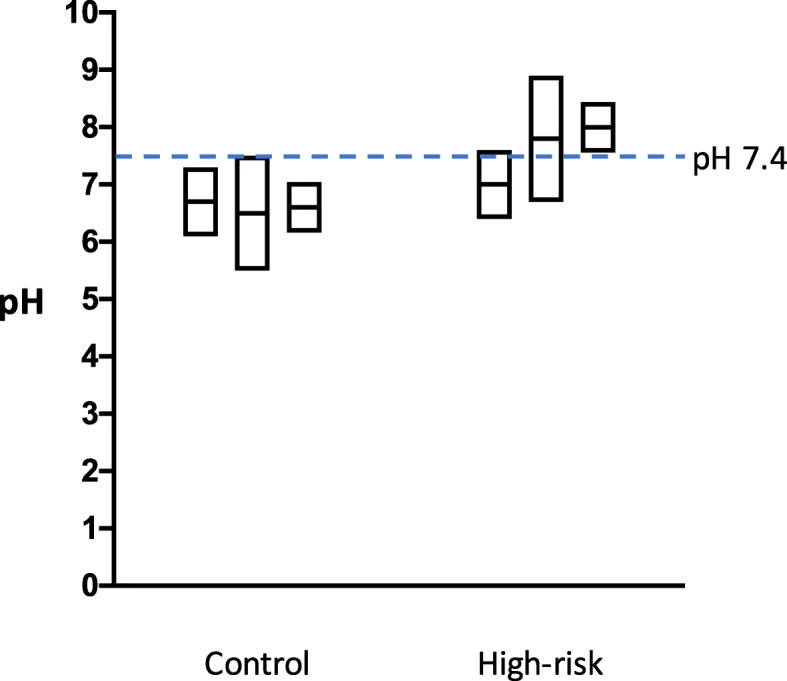
Comparison of fecal pH ranges between control and colon cancer high risk groups

In normal subjects, the stomach fluid pH range is 1.1–2.1 under fasted conditions, and 1.0-5.6 after a meal, as shown in Fig. 9 [28, 29]. On the other hand, in the high risk gastric cancer groups, such as achlorhydric patients, and especially those with pernicious anemia or hypogammaglobulinaemia, or subjects with gastric ulcers associated with *H. pylori* infection, the stomach fluid approaches a neutral pH (4.0-8.3) [30–32]. The gastric cancer risk is significantly enhanced by the interaction of red meat consumption and *H. pylori* infection (presumably due to high gastric pH) [33, 34]. The pH values of fasted and postprandial gastric juices in high-risk groups seem to be similar, as determined previously [28]. Differences in fecal pH (5.5–7.6 versus 6.4–8.9) were also observed between the control and colon cancer high risk groups (Fig. 10) [35]. In the present study we chose pH 3.5, 4.5 and 7.4 as representative pH values for the model reactions, considering the pH values of the normal and high-risk groups. Enhanced AcAld formation was observed especially at pH 7.4 in the hemin-MLA-wine and salami-MLA-wine reactions, as compared to the ethanol-containing reaction. The potent enhancing effect at pH 7.4 may be due to the generation of H_2_O_2_ by the autooxidation of wine polyphenols at neutral pH, as in many other plant polyphenols [36]. The time-dependent formation of AcAld in a model reaction of H_2_O_2_, Mb and EtOH at pH 7.4 supported this mechanism (Fig. 5). H_2_O_2_ production may also occur in the colon, because the colorectal mucosal surface is usually aerobic due to oxygen diffusion from the colorectal submucosa [37]. The polyphenols in red wine and coffee also stimulated AcAld formation at pH 3.5 with baked meat. This may be due to the generation of H_2_O_2_ with the concomitant reduction of MetMb(Fe^3+^) to Mb(Fe^2+^), which would enhance OH-radical generation and AcAld formation (Scheme). In the present study, the effect of coffee was examined at a normal gastric pH (3.5). It is highly possible that the enhanced AcAld formation by coffee consumption may also occur at neutral pH. Most orally ingested polyphenols (90–95 %) reportedly reach the colon [38]. Therefore, the high AcAld formation in the meat- and heme-reactions with wine or coffee may be related to both gastric and colorectal carcinogenesis.

In a previous study, we found that baked meat has lower AcAld forming activity than raw meat and salami, with MLA and ethanol at pH 7.4. In this study, coffee enhanced AcAld formation in the mixture of baked meat and red wine at pH 3.5. Even with low concentrations of baked meat homogenates, red wine, and coffee, the AcAld level increased to 170 µM in 5 h (Fig. 4). The amounts of meat and wine in this model reaction correspond to 24 g meat and 83 ml each of wine and coffee, if the gastric digestion volume is tentatively 500 ml and the Mb content in meat is 1 %. Therefore, the observed AcAld formation in the reaction under-estimated the actual dietary situations, because usually the amounts of meat, wine and coffee in the diet are much higher. In the salami + wine experiments, where the AcAld concentration reached 540 µM (Fig. 2), the amounts of salami and wine corresponded to 50 g and 120 ml per 500 ml, respectively.

AcAld has been detected in human feces by GCMS, as a potential diagnostic tool for gastrointestinal diseases [39]. Urinary volatile organic compounds including AcAld have been analyzed as possible markers of colorectal cancer [40]. However, in those studies, only the GCMS patterns were compared between healthy people and patients, and the AcAld concentration was not monitored. Therefore, the present study is the first quantitative analysis of human fecal AcAld. Although the increase in the fecal AcAld concentration detected in this study was quite low (around 4 µM), the Meat + Wine diet showed a significantly higher level of AcAld, as compared to the Fish + Wine and Meat + Non-alcohol diets (Fig. 8). The small increase of AcAld may have occurred because the meat-rich diet in this study is within the range of a healthy diet containing vegetables and fruits, and the amounts of meat and wine were not very large; namely, 150–200 g meat and 120–150 ml red wine per day. In other words, with a balanced diet containing meat, wine, vegetables, fruits and coffee, the AcAld level does not reach the minimum mutagenic concentration (50–100 µM) [13].

The first pass metabolism (FPM) of ethanol to AcAld in the stomach (40–50 % of oral ethanol) before reaching the liver has been discussed [41, 42]. However, it remains difficult to explain the FPM of ethanol by enzymatic conversion, because the total ADH activity in the stomach is insufficient to account for FPM. Cortot et al. reported that enhanced alcohol disappearance (73 % of oral ethanol dose) occurs in the stomach, after a meal containing meat [43]. When alcohol was ingested orally after fasting, or by intravenous (iv) infusion, the FPM was small [44]. Therefore, the free radical-mediated mechanism of ethanol to AcAld conversion with a meat diet provides a possible answer to explain the FPM phenomenon in the stomach.

In relation to gastric cancer, these model reactions probably occur in the stomach, because the pH of the reaction mixture and the concentrations of Mb/Heme, wine and linoleate are comparable to those in gastric juice after a meal. In addition, the AcAld formed in gastric juice has no chance of clearance by ALDH, and may accumulate over a long period. Regarding colorectal cancer, a 13-fold greater amount of OH radicals is reportedly generated in human feces after a high meat and high fat diet with few vegetables, as compared to that after low meat and low fat diet with sufficient vegetables [45]. Persistant colonic ethanol, which is maintained for several hours after drinking [46, 47], may react with the OH radical generated in the colon to form AcAld. Therefore, the analysis of fecal AcAld levels may be a promising early marker of colon cancer risk. Further molecular epidemiological studies are required to clarify the relationships between dietary habits, such as high red meat, wine and coffee consumption, with and without fruits and vegetables, the AcAld levels in gastric juice and feces, and cancer induction.

## Conclusions

Acetaldehyde (AcAld) is generated from Heme/Mb/Meat-Linoleate-EtOH/Wine model reaction mixtures by a free radical mechanism. Depending on the conditions, such as pH, reaction time and choice of free hemin, myoglobin (Mb), or meat extracts (raw meat, baked meat, salami), wine and coffee enhanced AcAld formation. The fecal AcAld level was increased by the consumption of red meat with wine.

## Data Availability

Not applicable.

## Abbreviations

AcAld: Acetaldehyde
MLA: Methyl linoleate
Mb: Myoglobin
ADH: Alcohol dehydrogenase
ALDH: Aldehyde dehydrogenase
FPM: First pass metabolism
